# The Morphometry of Lake Palmas, a Deep Natural Lake in Brazil

**DOI:** 10.1371/journal.pone.0111469

**Published:** 2014-11-18

**Authors:** Gilberto F. Barroso, Monica A. Gonçalves, Fábio da C. Garcia

**Affiliations:** 1 Department of Oceanography and Ecology, Federal University of Espírito Santo, Vitória, Espírito Santo, Brazil; 2 Espírito Santo State Water Resources Agency, Vitória, Espírito Santo, Brazil; NERC Centre for Ecology & Hydrology, United Kingdom

## Abstract

Lake Palmas (A = 10.3****km^2^) is located in the Lower Doce River Valley (LDRV), on the southeastern coast of Brazil. The Lake District of the LDRV includes 90 lakes, whose basic geomorphology is associated with the alluvial valleys of the Barreiras Formation (Cenozoic, Neogene) and with the Holocene coastal plain. This study aimed to investigate the relationship of morphometry and thermal pattern of a LDRV deep lake, Lake Palmas. A bathymetric survey carried out in 2011 and the analysis of hydrographic and wind data with a geographic information system allowed the calculation of several metrics of lake morphometry. The vertical profiling of physical and chemical variables in the water column during the wet/warm and dry/mild cold seasons of 2011 to 2013 has furnished a better understanding of the influence of the lake morphometry on its structure and function. The overdeepened basin has a subrectangular elongated shape and is aligned in a NW-SE direction in an alluvial valley with a maximum depth (Z_max_) of 50.7****m, a volume of 2.2×10^8^ m^3^ (0.22****km^3^) and a mean depth (Z_mv_) of 21.4****m. These metrics suggest Lake Palmas as the deepest natural lake in Brazil. Water column profiling has indicated strong physical and chemical stratification during the wet/warm season, with a hypoxic/anoxic layer occupying one-half of the lake volume. The warm monomictic pattern of Lake Palmas, which is in an accordance to deep tropical lakes, is determined by water column mixing during the dry and mild cold season, especially under the influence of a high effective fetch associated with the incidence of cold fronts. Lake Palmas has a very long theoretical retention time, with a mean of 19.4 years. The changes observed in the hydrological flows of the tributary rivers may disturb the ecological resilience of Lake Palmas.

## Introduction

Lake morphology has been recognized as a key factor for the understanding of lacustrine structure and function. Since the late 1930s, based on Rawson’s diagram [1], lake area and depth contours have been viewed as factors controlling ecosystem productivity due to light penetration, heat balance, oxygen distribution, the input of allochthonous matter, the nature of the sediments and littoral zone development. The influence of the relative shape and size of lake basins on several lake processes has been investigated. These processes include mixing dynamics [2]–[4], hydrology [5], sedimentation [6], dissolved organic carbon content [7], the biomass of submersed macrophytes [8], primary productivity [9] and lake metabolism [10].

Lake morphology is quantified with morphometric metrics that are descriptors of the form and size of lake basins. This analysis provides crucial knowledge in support of approaches to lake management. Geographical information systems are becoming an important tool to process and analyze morphometric metrics of areas and volumes [11]–[13].

Geographical location of lakes (latitude, longitude and altitude) must also be considered due to the climatic drivers of insolation, wind and precipitation. Lake typologies for many different geographical settings have been established based on lake morphology and climate [14], [15].

In Brazil, with the exception of the extensive study of 61 coastal lakes in the southern portion of the country [16], lake morphometric studies are relatively scarce. Morphometric data are generally available for artificial lakes, particularly in terms of reservoir engineering and management [17]. The deepest natural lake in the country for which data have been published is Dom Helvécio, in the Middle Doce River Valley - MDRV (State of Minas Gerais, Southeastern Brazil), with maximum and mean depths of 39.2 and 11.3 m, respectively [18]. This well-known lake, whose genesis is fluvial, shows a warm monomictic pattern. The lower Doce River Valley (LDRV) (State of Espírito Santo, Southeastern Brazil) has 90 lakes, comprising a valuable water resource that needs sound environmental management.

The present study aims to improve the ecological knowledge of moderate tropical deep lakes through the determination of several morphometric factors for Lake Palmas. A geographic information system (GIS) was developed as an environment for metrics calculation. Wind climate effects on lake stratification and mixing, based on wind direction and intensity, were also integrated in the GIS approach. Vertical water column profiles were developed to explore the relationships between lake morphology and water column stratification.

## Materials and Methods

No specific permissions were required to collect hydrographic data and temperature and dissolved oxygen data in Lake Palmas (19°23′S/40°17′W and 19°26′S/40°13′W). In addition, field and lab studies did not involve any biological species.

### Physiography of the study area

**Figure 1:**
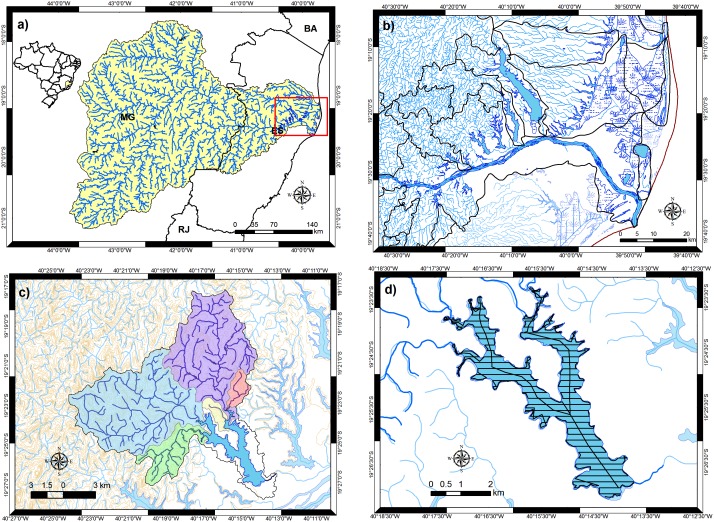
Study area settings: a) LDRV location on the southeastern coast of Brazil in the Doce River Basin (State of Espírito Santo); b) the LDRV Lake District, with its “lakescape”; c) Lake Palmas and its watershed and height curves; d) echosounding transects for hydrographic survey of Lake Palmas.

The LDRV is the location of a district including 90 lakes with areas ranging from 0.8 ha to 62.0 km^2^, a total lake area of 165.5 km^2^ (Figure 1a and 1b). The LDRV Lake District and its ‘lakescape’ comprise lakes located in dammed alluvial valleys and lakes located on the coastal plain. According to Bozelli et al. [19], the lakes of the LDRV show both intermittent and dynamic patterns of metabolism. The intermittent pattern is found in the lakes of the alluvial valleys of the Barreiras Formation (Cenozoic, Neogene Period), which are functionally deep and can be described by a seasonal metabolism model. The dynamic metabolism pattern is characteristic of the coastal plain lakes (Cenozoic, Quaternary Period, and Holocene Epoch), which are relatively shallow and are more efficient in processing organic matter.

In the easternmost part of the LDRV, neotectonic processes with patterns of alignment from the NW to the SE control the drainage system of the major river, the Doce River, and its tributary rivers and lakes in the alluvial valleys [20]. According to Martin et al. [21], the Doce River delta and the associated Holocene sedimentation of an ancient lagoon represent a breakthrough process of regional geomorphologic evolution. This process, which started approximately 5,100 yrs B.P., is associated with sea level transgression and regression on the paleodeltaic coastal plain and the damming of the alluvial valleys of the Barreiras Formation with fluvial sediments [21].

The regional climate is characterized by relatively wet and hot summers and dry/mild cold winters. Land use is dominated by pastureland, croplands and *Eucalyptus* forestry. The major areas of urbanization are located in the southeast portions of the District, in the vicinity of Juparanã (62.0 km^2^), Meio (1.3 km^2^), and Aviso (0.7 km^2^) Lakes [22]. In general, lakes water resources have been used for crop irrigation, recreation, fishing and, more recently, for aquaculture. There are two intensive fish farming operations in Lake Palmas, which produce tilapia in floating cages.

### Hydrographic survey

The Lake Palmas (Figure 1c) shoreline was screen digitized with the geographic information system ArcGIS 10.1 ESRI (Redlands, California, USA), software licensing EFL615216336, using a digital aerial orthophotograph acquired on May 2008 at a scale of 1∶15,000 and a spatial resolution of 1 m, georeferenced with Universal Transverse Mercator (UTM) projection zone 24 k and World Geodetic Datum (WGS) 1984. The polygon shapefile of the lake surface retained the projection and datum of the orthophoto. Echosounding survey lines were plotted on the polygon shapefile at a spacing of 200 m along the longitudinal axes of the lake (Figure 1d). A total of 100.7 km was selected for bathymetric sounding.

A hydrographic survey was performed in May 2011 with an Ohmex (Sway, Hampshire, UK) HydroLite XT echosounding system composed of a 210.0 kHz single beam transducer, a SonarMite V3 BT Bluetooth connection and a Trimble (Sunnyvale, California, USA) GeoXH DGPS receiver. Navigation along the survey lines was oriented with the shapefile of transects displayed with ArcPad 7.0 ESRI on a Trimble Juno GPS receiver. Spatial information was determined according to the Universal Transverse Mercator (UTM) projection and World Geodetic Datum (WGS) 1984. Lake hydrographic surveys were performed at a maximum boat speed of 5.0 km.h^−1^ (2.7 knots).

### Bathymetric data processing

X, Y and Z (easting, northing and depth) data were downloaded to Ohmex SonarVista software and then exported as a *dxf* file. In ArcGIS 10.1, the *dxf* files were converted to point features in a shapefile format. The attribute table of the shapefile of depth values was edited to identify and erase depth spikes. The lake shoreline shapefile was converted from a polygon to a point file with a yield of 1,539 shoreline points, which were assigned to zero depth. This shapefile was later merged into the bathymetric survey shapefile.

The interpolation procedure for generating a surface model of the bathymetry data was conducted with Ordinary Kriging using the ESRI extension Geostatistical Analyst 10.1. The process is based on semivariogram modeling, neighborhood search and crossvalidation [23], [24]. The resulting bathymetric map was presented with 5.0 m isobaths.

The intensity of the survey (L_r_) is the ratio between the lake area in km^2^ and the echosounding track length in km. The accuracy of the bathymetric map was assessed with the information value (*I*), which indicates a completely correct map when *I* = 1. *I* was calculated as a product of correctly identified area (*I’*) and information number (*I”*). *I’* and *I”* also vary between 0 and 1, with a value of *I’* = 1 indicating that all contour lines are correct and a value of *I”* indicating that the number of contour lines is optimal. The equations for *I*, *I’* and *I”*, given by Håkanson [11], [25], incorporate lake area (km^2^), the distance between the sounding tracks (km), shoreline development and the number of bathymetric contour lines. The symbols for the morphometry metrics are based on Hutchinson [26].

### Lake size metrics

The maximum depth (Z_max_) was determined from the echosounding points after editing to remove spike data. Lake perimeters, areas, and volumes were calculated with ArcGIS 10.1 routines to determine primary morphometric parameters for lake size, such as lake surface area - A (m^2^), shoreline length - L_0_ (m), maximum length - L_max_ (m), maximum breadth - B_max_ (m) and volume V (m^3^).

### Lake form metrics

Lake form factors were calculated according to Håkanson [11] as follows: mean depth in m, 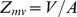; relative depth in %, 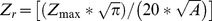; shoreline development index, 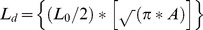; volume development, 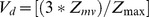; and mean basin slope in %, 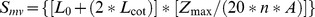.

Where, V = lake volume in m^3^, A = lake water surface area m^2^ in (in km^2^ for S_mv_), Z_max_ = maximum depth in m, L_0_ = normalized shoreline length in km, L_ctot_ = total normalized length for all contour lines in km excluding the shoreline and *n* = number of contour lines.

### Water column structure

**Figure 2:**
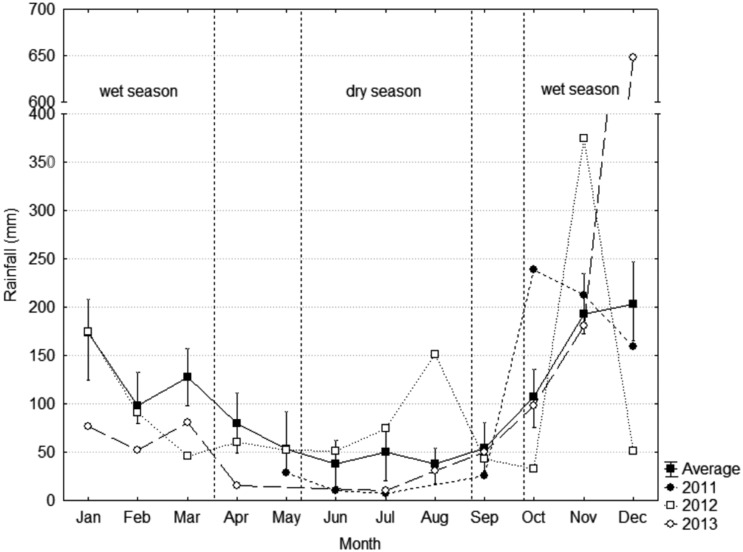
Regional mean monthly rainfall (1947 to 2013) and mean monthly rainfall for the study period (2011 to 2013). Data from 13 meteorological stations (National Water Agency – ANA).

Water column profiles for temperature (°C), photosynthetically active radiation (PAR) and dissolved oxygen (mg.L^−1^) were recorded for the field samples from 2011 to 2013 in wet/warm months and in dry/mild cold months. Based on data from 1947 to 2013 from 13 meteorological stations (National Water Agency – ANA, hidroweb.ana.gov.br), the wet months show a regional mean monthly rainfall greater than 100 mm, whereas the regional mean monthly rainfall for the dry months is less than 50 mm (Figure 2). The wet/warm season extends from October to March, with a mean monthly rainfall of 167.6±32.2 mm and a mean air temperature of 24.8±3.25°C. The dry/mild cold season extends from May to August, with a mean monthly rainfall of 46.1±2.5 mm and a mean air temperature of 21.9±3.1°C.

**Figure 3:**
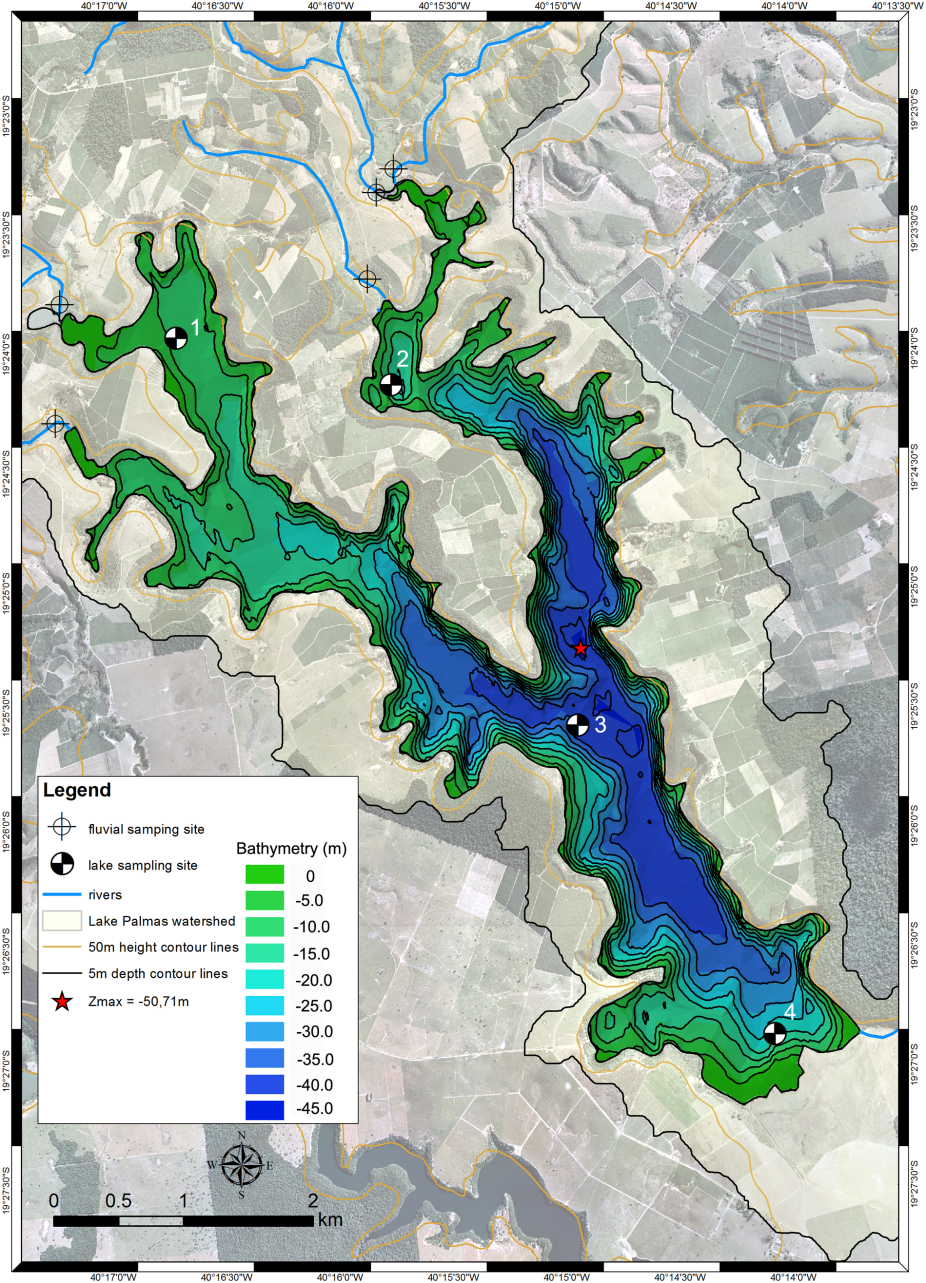
Lake Palmas bathymetric map.

Water transparency was estimated with the depth of Secchi disk. A Horiba (Minami-Ku, Kyoto. Japan) U-53G multiparameter water quality meter with a 30 m cable was used for vertical profiling at 4 sampling sites along the lake axes (Figure 3). Bottom water samples were taken with a Niskin bottle. The extent of the euphotic zone (Z_eu_) was estimated with underwater light attenuation to a depth corresponding to 1% of subsurface PAR through vertical profiling with a LiCor (Lincoln, Nebraska USA) system with a LI-250A light meter and LI-193 spherical PAR quantum sensor.

The mixing depth (Z_mix_) in m was calculated based on the maximum discontinuity in the relative thermal resistance (RTR) [27]. The thermal resilience of the water column, based on the Effective Wedderburn number (W_e_) [28], [29], was calculated for each sampling site at every field sampling event:
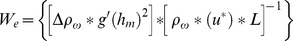
where, *Δρ_ω_ = *the difference between the water mass density at the upper and lower limits of the thermocline (kg.m^−3^), *h_m_ = *mixing depth (m), *L* = effective fetch in m, g’ = the reduced gravity and u* = the wind friction velocity, g’ and u* are calculated with the following equations:



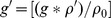
where, g’ is the reduced gravity, g is the normal gravitational acceleration, *ρ*’ is a density perturbation and *ρ*_0_ is a standard reference density;



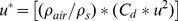



Where, *ρ_air_* is the air specific mass in kg.m^−3^, *ρ_s_* is the water specific mass at the lake surface in kg.m^−3^; *C_d_* is the coefficient of friction (0.0015) and *u* is the wind velocity in m.s^−1^. If *W*>1, the thermal structure is stable; if *W*<1, the water column is susceptible to changes resulting from the effects of wind.

Hourly wind direction and intensity data were obtained from observations at the Linhares meteorological station (INMET - A614), approximately 18.0 km NE of Lake Palmas.

### Special metrics for lake morphometry

The A/V ratio was calculated to estimate the potential evaporation rate of lake water and the resistance of the water column to mixing. The slope of the lake basin was modeled in terms of the percent rise function using ArcGIS 10.1 with the function Slope, 3D Analyst Tool. The continuous surface model for the basin slope was reclassified in terms of gentle and steep slopes. According to Duarte and Kalff [8], a slope of 5.3% is the threshold value separating gentle and steep slopes in relationship to the development of submersed rooted aquatic vegetation in lakes.

The wave base depth (Z_wb_) in meters was calculated from the 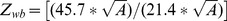, with A in km^2^ [11]. Z_wb_ was the depth used to estimate the volume of the epilimnetic waters. The delimitation of the littoral and pelagic zones and their respective volumes was performed based on the mean depth of the euphotic zone (Z_eu_) in meters. The area suitable for the development of rooted aquatic vegetation biomass was also based on the mean Z_eu_ and within gentle slopes (<5.3%). Volumes in m^3^ for hypoxic/anoxic bottom waters were determined based on the depth corresponding to hypoxia (<2.0 mg.L^−1^) during the stratification season.

The effective fetch, L_ef_ (km) and the wave heights (m) were estimated for 46 sites distributed along the lake surface with a grid with equal distances of 500.0 m. To estimate L_ef_, distances from each site to the shoreline were measured according to the prevailing winds (defined as an angle of 0°) and every 6° on both sides of the 0° angle to 42° [11]. The below provides the integrated value of L_ef_:
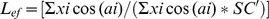
where, *Σcos(a_i_) is* 13.5, a calculation constant, and *SC’* is the map scale constant of 0.35.

Wave heights (H) in m were computed for the sites for which L_ef_ was estimated, according to the Beach Erosion Board (1972) in Håkanson [11] using the following equation:
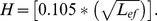



Both *L_ef_* and H were calculated based on two prevailing wind directions, one for the wet/warm season (NE) and one for the dry and wet season (SE, considering the major axis of the lake). Maps of L_ef_ and H maps were created using GIS, interpolating the point data with a spline function with tension, a neighborhood of 5 points, a weight of 0.01 and a cell resolution of 5.0 m.

The basin permanence Index (BPI) (m^3^.km^−1^), which indicates the littoral effect on basin volume, is calculated according to the ratio of lake volume (×10^6^ m^3^) to shoreline length (km), 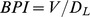 [30]. The dynamic ratio (DR) was calculated according to the equation: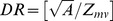 with A in km^2^ [11].

To assess the cryptal depth (Z_c_) and cryptal volume (V_c_) of the lake, depth values were converted according to the altitude of the lake surface above sea level, using an altitude of 20 m as the reference value for the lake surface.

The theoretical lake water retention time was calculated according to the ratio of the lake volume (m^3^) to the mean annual river tributary inflow (m^3^.s^−1^) 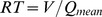 [41], [42]. Discharge of the five tributary rivers (Figure 3) were measured during the wet/warm and dry/mild cold seasons (n = 8) with a SonTek (San Diego, California, USA) FlowTracker Handheld Acoustic Doppler Velocimeter (ADV). Mean annual river tributary discharges were then calculated, as well as discharge values for dry and wet seasons.

## Results

The total bathymetric sounding survey track was 122.9 km, yielding a survey intensity (L_r_) of 0.08. A total of 46,941 valid depth points were computed. Ordinary kriging to obtain prediction results was applied as the interpolation method to yield a continuous surface of lake depths. A neighborhood search was used, considering a smooth type within an axis range between 100 to 2,000 points. Variogram modeling was based on 9 lags with a size of 280 and a spherical model with anisotropy (a direction of 125°). The regionalized variation of point data, optimized sampling and spatial pattern determination is addressed with the Semivariogram on Figure S1. The bathymetric map, with a cell size of 10×10 m (Figure 3).

Based on depth contour intervals of 5 m, the correctly identified area (*I’*) is 0.8571. This value means that 85.7% of the lake area was correctly identified and that 14.3% (1.5 km^2^) of the lake area was incorrectly estimated. The information number (*I”*) for the 5 m contour lines was 0.9995, and the information value (*I*), indicating the overall map accuracy, was 0.8566.

The lake basin has a ‘Y’ shape aligned in a NW-SE direction, with a maximum length (L_max_) of 7.1 km and an average breadth (B_mv_) of 1.7 km. Other lake basin size metrics were a total surface area of 10.3 km^2^, a shoreline length (L) of 51.9 km, a maximum depth (Z_max_) of 50.7 m and a volume of 2.2×10^8^ m^3^ (0.2 km^3^).

The lake form metrics were found to have the following values: the shoreline development index (D_L_) was 4.5, the mean depth (Z_mv_) was 21.4 m, the relative depth (Z_r_) was 1.4%, the volume development (V_d_) was 1.3, the mean slope (S_mv_) was 15.8% and the Z_mv_:Z_max_ ratio was 0.42. These metrics indicate a flat-bottomed, overdeepened lake basin. Based on the value of D_L_, the shoreline form is subrectangular elongate. The basin form is linear (L) according to the relative hypsographic area and volume curves (Figure S2a and S2b) as well as according to V_d_.

There are 18 embayments along the lake axis, most of which are less than 15.0 m in depth. The area deeper than 40.0 m extends from the intersection of the two lake axes to the S shore and to the upper N axis. The three major deepest basins (>45.0 m) are located midway on the N-S axis next to the intersection of the two lake axes, representing an area of 1.5×10^5^ m^2^ (1.5% of the lake area) and a volume of 5.1×10^5^ m^3^ (0.02% of the lake volume).

The lake drainage is also oriented from NW to SE, with 5 tributary streams located along the upper NE and N shores. Lake Palmas discharges into the Doce River through a drainage river located along the SW shore.

**Figure 4:**
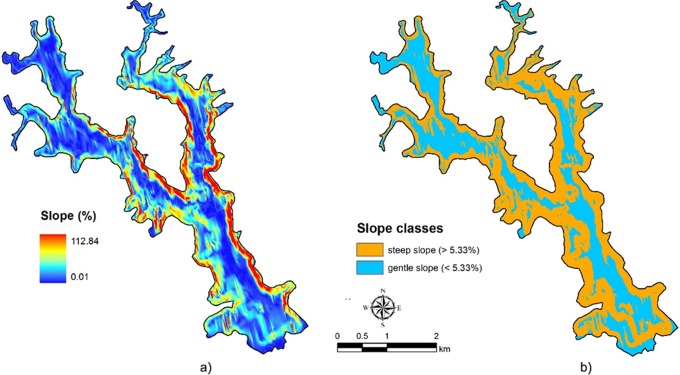
Lake bottom slope (%): a) slope gradient in %; b) reclassified slope: gentle (<5.33%) and steep (>5.33%) slopes.

The area/volume ratio is 0.05, indicating a deep basin with a small littoral zone. The mean basin slope (S_mv_) of 15.8% represents an overall value for the shallow areas, central basin plain and steep lateral slopes. The basin slope GIS model (Figure 4a) shows steep lateral slopes, up to 112.8%, along the central E shore as well as along the E and W shores of the promontory that separates the lake into two arms. The central basin plain is constrained with slopes lower than 20% and depths greater than 30 m. Shallow areas with a depth of less than 5 m and a slope of 5% are located at the mouths of the tributaries as well as the southernmost part of the lake. Very gentle slopes up to 2.0% are characteristic of deep basins (>40 m deep). Based on a threshold of 5.3%, in which fine sediments are retained [8], [11], 9.1 km^2^ (58.8% of the lake area) and 4.3 km^2^ (41.3%) were classified as steep and gentle slopes, respectively (Figure 4b).

**Figure 5:**
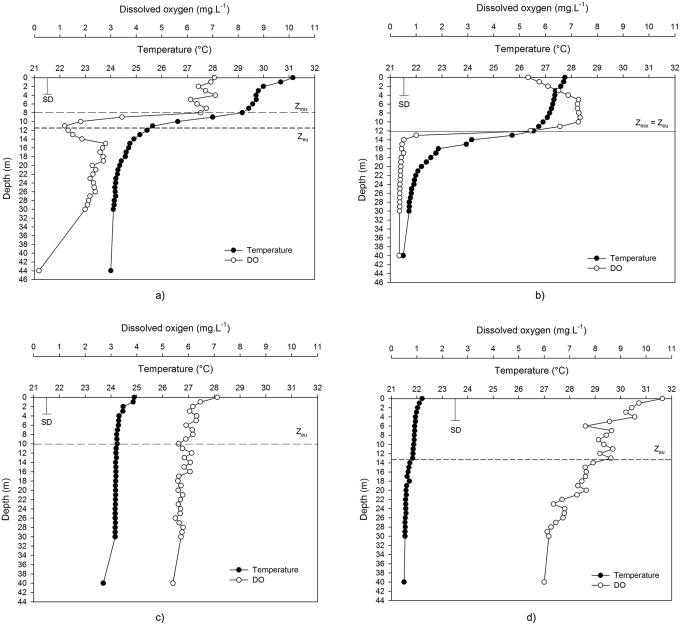
Typical vertical profiles of temperature and dissolved oxygen with mixing (Z_mix_) and euphotic (Z_eu_) zones and Secchi disk depth (SD) at the deepest Lake Palmas sampling site during the wet/warm (a and b) and dry/mild cold (c and d) seasons: a) 03/07/12, b) 03/12/13, c) 07/24/12 and d) 09/04/13.

Data from field vertical profiling at 4 sampling sites (Table S1) show stratification during the warm months, with surface water temperatures reaching 31.3°C (Figure 5a), and a mixed water column during the mild cold months, with a mean water temperature of 23.2°C. The Effective Wedderburn (W_e_) values, an overall indicator of the thermal stability of the water column, were 6.5±7.2 during the wet/warm season and zero in the dry/mild cold season. During the dry season, the mean Z_mix_ value of 20.7±8.3 m indicated the presence of a deep mixing layer but with a very weak, i.e., unstable, stratification due to the zero W value and an RTR lower than 3.1.

During the season of stratification, Z_mix_ was usually shallower or at least equal to Z_eu_ (Figure 5a), yielding higher Z_eu_:Z_mix_ ratios. In contrast, the Secchi disk depth (Z_Sd_) was higher during the mixing season. Based on a mean Z_eu_ of 10.0 m, the volume of the euphotic layer (V_eu_) was 9.2×10^7^ m^3^ (41.4% of the lake volume), whereas the volume of the aphotic zone was 1.3×10^8^ m^3^ (58.6%).

During the stratification season, hypoxic/anoxic conditions may develop below a depth of 13 m (Figure 5b). Under these conditions, the volume of anoxic waters may reach 1.1×10^8^ m^3^ or 48.6% of the lake volume. Bottom hypoxia/anoxia was recorded during the entire wet/warm season. In contrast, DO is well distributed in the water column throughout the mixing season (Figure 5c), even showing supersaturation at the surface (Figure 5d).

Special morphometry metrics show that the lake basin has a wave base depth (Z_wb_), an indicator of the depth of turbulent mixing, of 6.0 m, with a surface layer volume (i.e., a mixing layer) of 5.8×10^7^ m^3^, or 26.1% of the lake volume. Thus, the volume of bottom waters was 1.6×10^7^ m^3^, or 73.9% of the lake volume. The dynamic ratio (DR) had a value of 0.15 indicating the predominance of slope processes over wind/wave processes in sediment resuspension.

**Figure 6:**
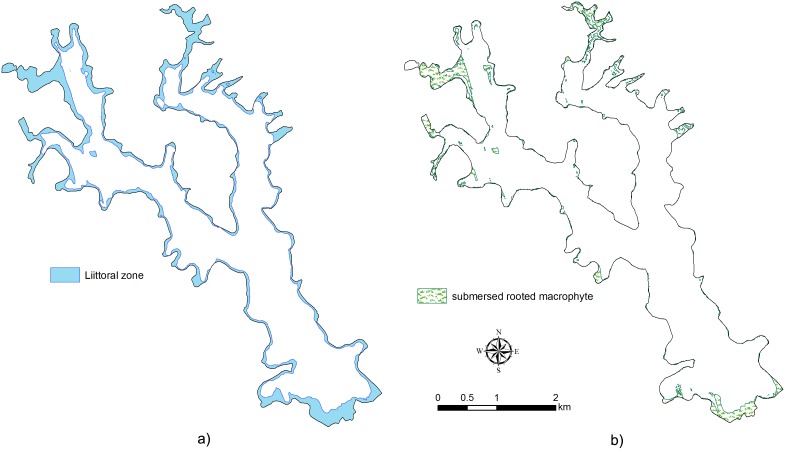
Lake littoral zone: a) littoral zone with a threshold of a depth of 10.0 m for the euphotic zone; b) potential submersed macrophyte area with a depth of 10.0 m or less and gentle slopes (<5.33%).

The Basin Permanence Index (BPI), 4.3 m^3^.km^−1^, indicates that Lake Palmas is relatively less suitable for the development of the littoral zone and rooted aquatic plants. With the same threshold of 10.0 m for Z_eu_, the littoral and pelagic zones are represented by 8.1×10^6^ (3.7% of the lake volume) and 2.1×10^8^ m^3^ (96.3%), respectively (Figure 6a). The predicted potential areas for rooted submersed vegetation with nearshore gentle slopes comprise only 7.9×10^5^ m^2^, less than 1.0% of the bottom area of the lake (Figure 6b).

**Figure 7:**
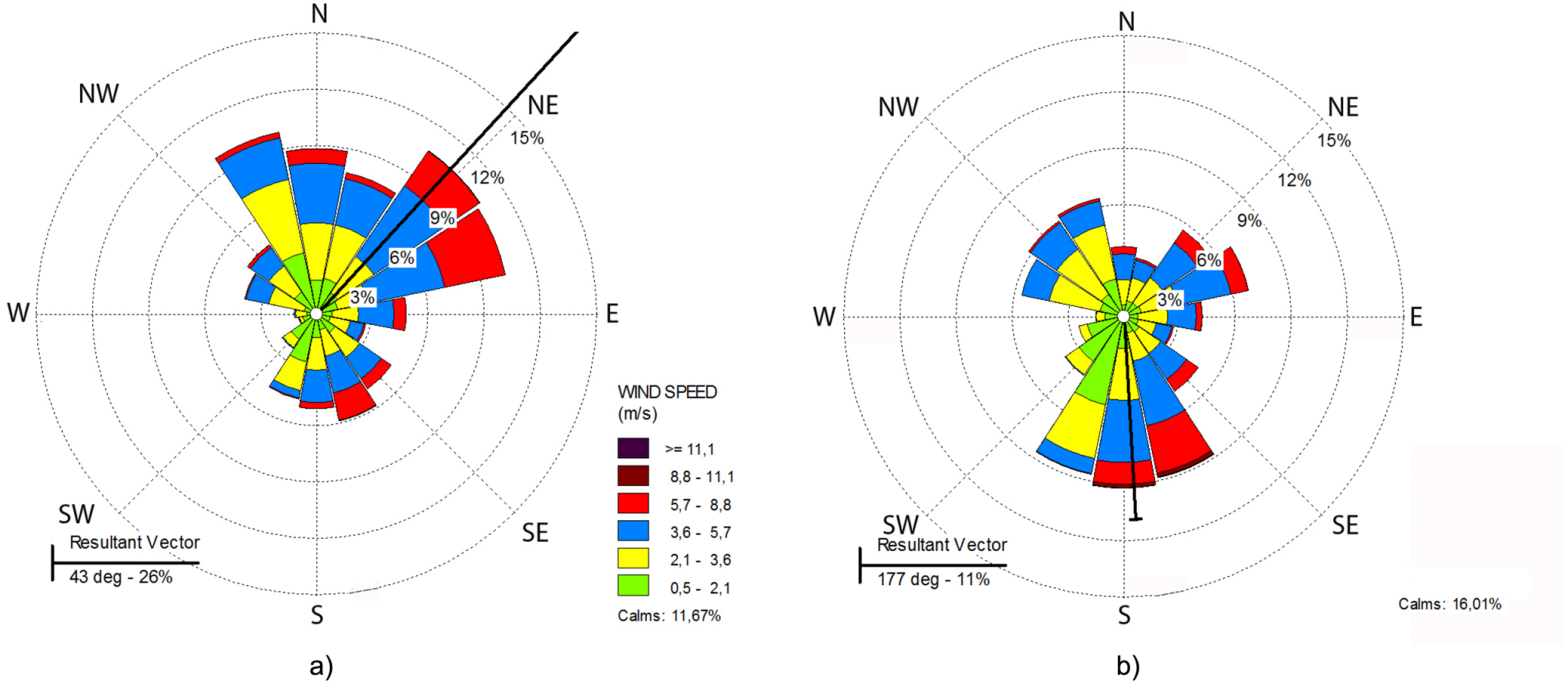
Wind direction frequency (%) and intensity (m.s^−1^) from Linhares meteorological station from 2007 to 2009: a) warm and wet months; b) dry/mild cold months.

Wind pattern for the warm/wet months showed a dominance of 26% from the NE, with wind speeds up to 8.8 m.s^−1^ (Figure 7a). During the dry/mild cold months, the wind was predominantly from the S, with speeds up to 11.1 m.s^−1^ (Figure 7b).

**Figure 8:**
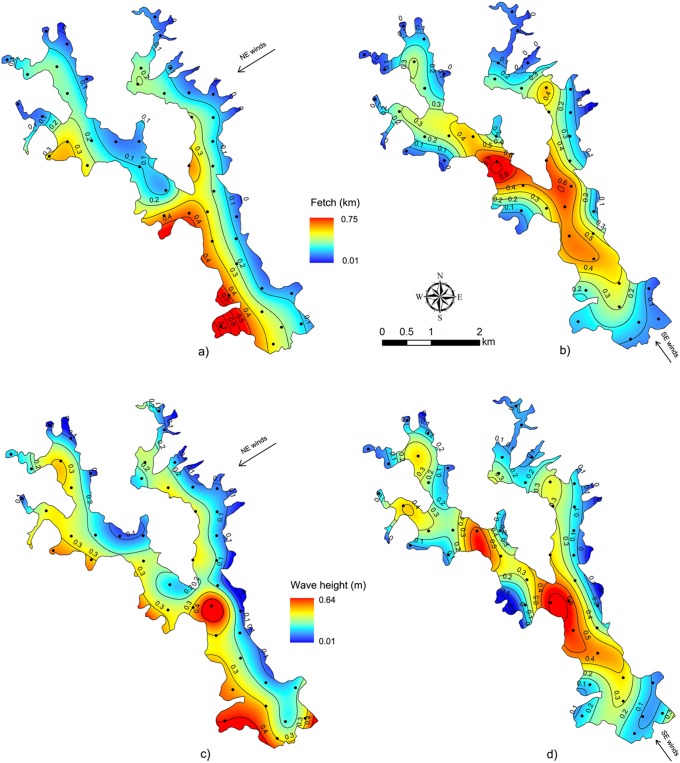
Effective fetch (L_ef_) and wave height (H) models. a) L_ef_ from NE winds; b) L_ef_ from SE winds; c) wave heights from NE winds; and d) wave heights from SE winds. Contour lines show 0.1 m and km intervals.

The effective fetch (L_ef_) model for wet/warm months with NE winds yielded values up to 0.8 km at the SW embayment (Figure 8a) and wave heights up to 0.5 m in the same embayment and at the confluence of the two axes (Figure 8c). For the dry/mild cold months, with SE winds, values up to 0.72 km were found at the lower section of the NW-SE axis and at the southern part of the land promontory (Figure 8b). Under SE winds, the wave heights were up to 0.6 m at the W shore next to the land promontory as well as in the central section of the NW-SE axis (Figure 8d).

**Figure 9:**
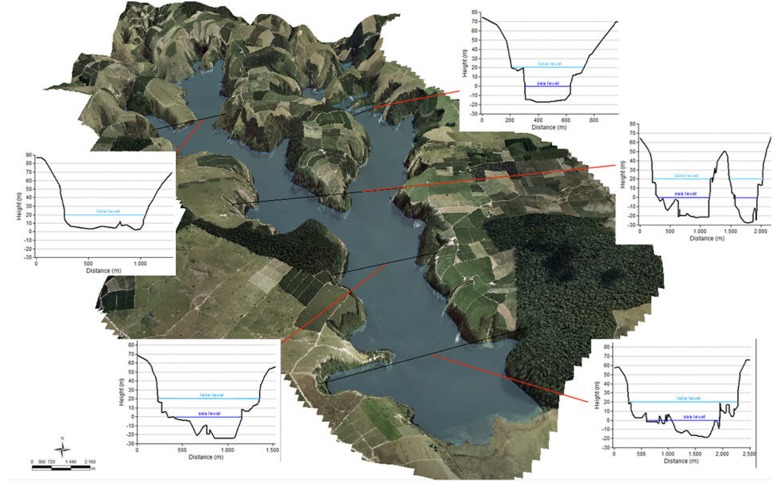
3D view of the terrain model and height profiles: the light blue line refers to the lake level, the dark blue line to sea level. The cryptal volume is the volume below the dark blue line.

As the lake surface is 20 m above mean sea level, the cryptal depth (Z_c_) is 20.0 m, with a corresponding cryptodepression volume (V_c_) of 2.8×10^7^ m^3^ (12.6% of the lake volume) (Figure 9). The water column is free from the influence of salt water.

The Lake Palmas watershed area (W_A_) is 168.2 km^2^, and the W_A_/A ratio is 16.3. The mean annual, dry/mild cold and wet/warm total tributary discharge values were 0.4±0.2, 0.3±0.3 and 0.4±0.04 m^3^.s^−1^, respectively. The river discharge during the wet/warm season was 10.0% higher than the annual mean. In contrast, the dry season discharge was 14.6% lower. Zero discharge was registered three times for tributary river 1 during the dry season, but tributary 5 dried up twice during the wet/warm season.

The theoretical retention time based on the mean annual tributary discharge was 19.4 years, which may increase or decrease up to 20.7 and 17.7 years, considering the low and high discharges of the dry and wet seasons, respectively.

## Discussion

According to the D_L_ criteria proposed by Hutchison [26], D_L_>2.5 and <5.0, the shoreline form of Lake Palmas is subrectangular elongated. Although this D_L_ range was thought to designate lakes in overdeepened valleys associated with tectonic grabens or glaciated fjords, the geomorphology of Lake Palmas is associated with fluvial erosional and depositional processes in alluvial valleys and with Holocene sea level transgressions and regressions [21]. The relatively deep valley, from elevations up to 70 m at the Barreiras Formation plateaus down to – 30 m below sea level (Z_max_ = −50.7 m), may be associated with neotectonic processes, with valley alignments along the NW-SE axis [20]. The neotectonic hypothesis has also been supported by geophysical studies at Lake Juparanã (62.0 km^2^) [32], the largest lake in the LDRV.

The high V_d_ and Z_r_ values indicate that the basin form of Lake Palmas is an overdeepened valley with a relatively flat bottom. The mean lake slope is moderate despite steep areas at the SE shore of the lake and around the S section of the land promontory at the confluence of the lake axes. In addition, the relative hypsographic area and volume curves indicate a linear basin, an intermediate profile between concave and convex basins. Nevertheless, the basin linear profile represents the major component of water storage for the pelagic volume (96.3%). This characteristic is supported by the low BPI value and the low A:V ratio, reinforcing the relatively deep morphology of Lake Palmas. These metrics also emphasize a low potential for lake water evaporation and a higher potential for water column resistance to mixing.

The basin slope influences the processes of sediment erosion, transport and deposition as well as macrophyte biomass. Based on the critical value of 5.3% used to differentiate gentle slopes from steep slopes, the areas with gentle slopes (below 5.3%) are characterized by the deposition of fine sediments and the thriving stands of rooted macrophytes. Nearshore steep slopes support erosion and transport processes and decrease macrophyte biomass [8]. The dynamic ratio (DR) of 0.15 indicates the predominance of slope processes in view of the threshold value of 0.25, with higher values indicating the predominance of resuspension from wind and wave action [11].

Another feature of deep basins is the significant volume of the aphotic zone (58.0% of lake volume), which may constrain the development and distribution of the photosynthetic biota. Consequently, the limited littoral zone (3.7% of lake volume) associated with gentle slopes (<5.3%) [8] restricts the habitat of rooted submersed macrophytes to less than 1.0% of the lake bottom area. The depth of 10.0 m, corresponding to 1.0 atm of hydrostatic pressure, is a threshold for the vascular system of angiosperms and defines the boundary of the lower infralittoral zone [33].

Based on published studies (Table S2), it seems that, Lake Palmas is the deepest natural lake in Brazil in terms of both Z_max_ and Z_mv_. In the light of the predominance of the fluvial and coastal geomorphological genesis of natural lakes in Brazil, these depths are remarkable. Lake Dom Helvécio (A = 5.3 km^2^), in the MDRV (State of Minas Gerais), was formerly considered the deepest natural lake in Brazil, with the following metrics: Z_max_ = 39.2 m, Z_mv_ = 11.3 m and V = 59.6×10^6^ m^3^ [18]. In the same Lake District of Lake Dom Helvécio, Bezerra-Neto, Briguent and Pinto-Coelho [34] determined Z_max_ and Z_mv_ values of 11.8 and 4.7 m, respectively, for Lake Carioca, with an area of 0.14 km^2^ and a volume of 6.7×10^5^ m^3^. Schwarzbold and Schâfer [16] conducted an extensive survey of 61 coastal lakes of southern Brazil and found a lake as large as 802 km^2^, with Z_max_ = 4.0 m and Z_mv_ = 2.5 m (Lake Mangueira), but Lake Figueira (7.1 km^2^) was found to have the highest Z_max_ and Z_mv_ values, 11.0 and 5.7 m, respectively. Recent hydrographic surveys conducted in other LDRV lakes determined Z_max_ values of 33.9, 31.6 and 22.1 m for Lakes Nova (A = 15.5 km^2^, D_L_ = 4.5 and Z_mv_ = 14.7 m), Palminhas (A = 8.8 km^2^, D_L_ = 8.1 and Z_mv_ = 14.2) and Terra Alta (A = 3.9 km^2^, D_L_ = 3.1 and Z_mv_ = 9.0), respectively [35]. Although Lake Juparanã has a surface area 6 times greater than that of Lake Palmas, the estimated Z_max_ is approximately 20.0 m. Even when considering Amazon lakes associated with fluvial processes in the floodplain, it seems that these lakes are shallower comparing to the ones of LDRV. For instance Lake Calado (A = 8.0 km^2^) [36] and Tupé (A = 0.6 km^2^) [51] show during Amazon River peak flooding Z_max_ of 12.0 and 6.0 m, respectively.

Z_wb_ is a functional depth that separates areas of sediment transport occurring through resuspension via wind turbulence from areas of sediment accumulation with no resuspension. The concept is also very useful for delimiting the boundary between surface (epilimnetic) and bottom (hypolimnetic) waters [11]. Considering the variability of Z_mix_ during the stratification season, 8.4±2.5 m, a Z_wb_ value of 5.9 m can serve as an effective criterion to measure the significance of the physical and chemical stratification of Lake Palmas during the stratification season. Based on Z_wb_, the epilimnion volume of Lake Palmas is only 26% of the lake volume. This value is consistent with the effects of the overdeepened basin on the resistance to mixing.

A moderate effective fetch (L_ef_) may deepen the thermocline down to 12.0 m in the thermally stable water column of Lake Palmas. High L_ef_ values usually occur with SE winds, which are characteristic of cold fronts. According to Marchioro [37], 3 and 16 cold fronts were recorded during summer and winter/spring 2011, respectively, for Vitória (ES), which is located 90 km south of Lake Palmas. These cold fronts, characterized by S, SW and SSE winds blowing up to 8.8 m.s^−1^, may produce an average air temperature decrease of 7.1°C relative to the previous day’s temperature. On average, cold fronts may persist up to 3.3 days. Tundisi et al. [38] have reported that the incidence of cold fronts may cause vertical mixing during the summer in reservoirs in Brazil.

The significant volume of hypoxic/anoxic bottom waters remains for the entire wet/warm season. This finding implies severe dissolved oxygen deficits and, as a consequence, the potential release of dissolved inorganic nutrients and heavy metals that are chemically bonded to the sediments. Nevertheless, Lake Palmas can be considered an oligotrophic ecosystem with a low phytoplankton biomass, e.g., a mean chlorophyll *a* value of less than 1.0 µg.L^−1^ [36]. Given that Z_eu_ reaches the metalimnion and hypolimnion layers, it may produce a suitable climate with low light and rich nutrients, supporting the cyanobacteria community. This maximum in metalimnion phytoplankton pigments has been reported for Lake Dom Helvécio [39]. Lake Palmas may also exhibit maximum metalimnetic conditions with relative low concentrations of chlorophyll *a*. Despite the value of Z_eu_:Z_mix_ indicates that light is not a limiting factor for phytoplankton growth during the stratification season.

These metrics describe a relatively deep basin that promotes physical and chemical seasonal stratification with strong environmental gradients and a warm monomictic pattern. Stratification may inhibit phytoplankton biomass, producing an oligotrophic state despite anoxic bottom waters. These findings agree with the concept of the intermittent metabolism of the overdeepened lakes of the LDRV [19].

Wind climate is a key driving force in the deepening of the mixing depth during the stratified season. However, this process depends on the angle of incidence of the wind on the aquatic surface, i.e., the fetch. S winds associated with the occurrence of cold fronts approaching from the S of the continent are frequent during the dry/mild cold months. These winds blow along the major axis of the lake, which is aligned with the NW-SE direction of the drainage network. This condition, associated with lower thermal radiation, is effective in breaking down the thermal stability and mixing the entire water column. Under these circumstances, the effective Wedderburn number has low values, indicating low thermal stability.

The W_A_/A ratio of 16.27 indicates a relatively large drainage basin and a potentially significant discharge of river water into Lake Palmas, although the seasonality of rainfall and the potential negative effects of water and land uses in the watershed may halt the flow of tributary rivers during the dry season. The year-round unregulated use of the waters of the lake and tributary rivers for irrigation may also change the water balance in the lacustrine basin. Land use in the lake watershed is predominantly allocated to pasture, agriculture and forestry, representing 62.4% of the watershed area, whereas forested areas occupy only 32.8% of the watershed area [22]. If 30 and 100 m buffer areas are considered along the tributary river network and along the shoreline of Lake Palmas, agroecosystems represent up to 76.8 and 79.7%, respectively, of the buffer areas.

Accurate theoretical retention time (RT) estimates should be based on best knowledge of hydrological flows of tributary rivers inputs, evaporation rate, groundwater exchanges, and water consumption rates, instead of the simple ratio of the inflow to lake volume. Only 0.5% of the total number of natural lakes are known morphologically and hydrologically [40] in contrast, estimates of RT data are usually available for reservoirs construction and management. As rules of thumbs, RT is longer in deeper basin, reservoirs show shorter RT than lakes, and surface outflow from the lakes compared to deep outflow from the reservoirs. Reservoir RT in general is less than a year, with a threshold for reservoir limnology below 200 days [41], [42]. The effect of flow is very significant for reservoir ecological structure and functioning, with RT >1 year show trend to stratification, eutrophication, anoxic bottom and recurrency/persistence of cyanobacteria bloom [42]. Blooms of cyanobacteria have been recorded in Funil reservoir, a tropical system in Brazil, despite the very short RT (annual mean of 41.5 days). High inputs of allochthonous nutrients from the reservoir watershed promote the eutrophic status of this artificial lake. The increase of the residence time, up to 80 days during the dry season, promotes spatial variability in the ecological structure of the reservoir along the river-dam axis [43].

The RT of 19.4 years for Lake Palmas can be considered very long, particularly for the regional wet climate (annual mean of 1,027 mm.y^−1^). In order to put it in perspective, and despite the lack of RT data for Brazilian natural lakes, Lakes in the Yunnan plateau, southwest of China, such as the oligotrophic Lakes Fuxianhu (A = 211.0 km^2^, Z_mv_ = 89.6 m and V = 18.9×10^9^ m^3^), Luguhu (A = 48.4 km^2^, Z_mv_ = 40.3 m and V = 1.9×10^9^ m^3^) and Cheghai (A = 77.2 km^2^, Z_mv_ = 25.7 m and V = 1.9×10^9^ m^3^) show RT of 35.5, 17.7 and 12.5 years, respectively [44]. These longer RTs imply in poorly flushed systems with negative correlations with total nitrogen and phosphorus and chlorophyll a. With such long RT of overdeepened lakes of Yunnan plateau are associated with oligotrophic systems, acting as a sink for inorganic and organic matter and with a delay response to additional nutrient inputs from lake watershed. In another hand, longer RT may imply in lack of resiliency after ecosystem distress from cultural eutrophication [44]. In addition to information about phosphorus and nitrogen loads input to the lake, knowledge of the theoretical residence time is a key factor for regulating the uses of Lake Palmas, such estimating lake carrying capacity for fish farming.

Hazards to water quantity and quality caused by pollution, silting and the introduction of exotic species may impair the ecological resilience of the lake. In addition, climate changes involving the intensification of extreme hydrological events (specifically, a predicted shortening of the rainfall season with fewer rainy days but with more intense and frequent storm events) [45] can be a major driver of shifts in lacustrine ecosystems in the LDRV. In December 2013, an extreme amount of regional precipitation, 650 mm of rainfall, 3 times the month mean rainfall at Linhares meteorological station, caused a major flood in the LDRV. Aditionaly, it must be considered a scenario of lake surface warming as a consequence of global warming. For deep lakes this scenario implies in an increasing loss of energy through evaporation, a deeper mixing layer, and an earlier summer stratification. These factors may lead monomitic lakes to turn into holo-oligomictic, with a complete vertical mixing occurring eventually in some years when stratification become weaker during winter [46].

Water uses in the lake watershed may also increase the stress on the theoretical retention time of Lake Palmas. Of the 5 tributary rivers, 2 showed no discharge at all at least 5 times. Rivers also became dry during the wet/warm season. These events might have resulted from river damming for irrigation purposes, given that 55 small reservoirs for irrigation purposes with an area up to 0.6 km^2^ have been mapped in the Lake Palmas watershed [22].

Climate, lake morphology, and edaphic factors have been considered key drivers for trophic status of lakes, including the overdeepen basins. However, human impact factors have also been recognized, in some cases, as the leading driver for cultural eutrophication. The intensification of land and water uses in lake watersheds highlight the urgent need to regulate these uses in order to maintain a healthy lake ecosystems. In addition, climate change effects on water balance and related threats to ecosystem resilience and water security must be recognized.

## Conclusions

The subrectangular elongated shape and the relatively overdeepened basin of Lake Palmas place most of the lake’s volume in the pelagic compartment. Approximately one-half of the lake’s volume is within the aphotic zone. The overdeepened basin promotes the physical and chemical stratification of the water column during the wet/warm months of the year. Under these conditions, only a small part of the lake volume is prone to mixing effects, and a large volume remains hypoxic/anoxic. During the dry/mild cold months, the predominance of S-SE winds, characteristic of the arrival of cold fronts, and the high effective fetch of these winds on the basin aligned along a NW-SE axis effectively promote the mixing of the lake’s water column. Thus, the thermal pattern of Lake Palmas is warm monomictic. This finding is consistent with the hypothesis of a pattern of intermittent metabolism in the overdeepened lakes of the LDRV. Based on published data, Lake Palmas seems to be considered the deepest natural lake in Brazil in terms of both its maximum and mean depths. Given the very long theoretical retention time of Lake Palmas, hydrological changes in tributary rivers may increase the retention time and foster water quantity and quality problems. There are warning signs that the water balance in the basin is under pressure due to the unregulated uses of water for the year-round irrigation of croplands.

## Supporting Information

Figure S1 **Semivariogram for kriging interpolation of point data to generate a continuous surface describing the lake depth measurements.**
(TIF)

Figure S2 **Hypsographic curves of percent total surface (a) and total volume (b).**
(TIF)

Table S1 **Descriptive statistics of limnological variables in wet/warm and dry/mild cold seasons (2011 to 2013).** Data from field vertical profiling at 4 sampling sites” from surface to the bottom.
(DOCX)

Table S2 **Morphometry of natural lakes in Brazil deeper than 6.0 m.**
(DOCX)
