# Supplementary figures and images for: The Morphometry of Lake Palmas, a Deep Natural Lake in Brazil
Source: PLoS One. 2014 Nov 18;9(11):e111469. doi: 10.1371/journal.pone.0111469 (PMC4236007; doi:10.1371/journal.pone.0111469)

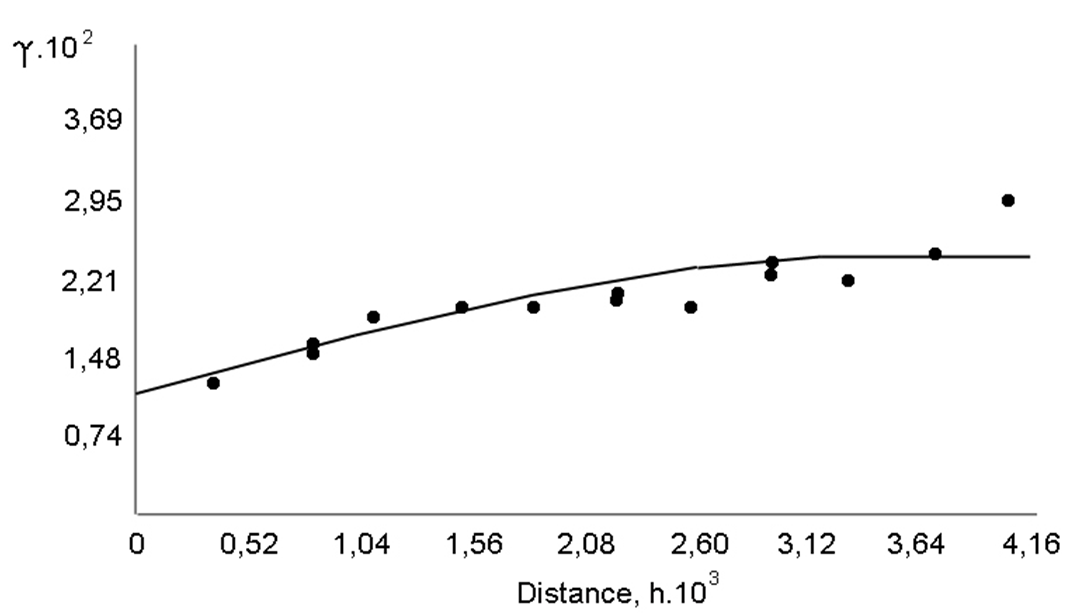

Supplement: Figure S1 — Semivariogram for kriging interpolation of point data to generate a continuous surface describing the lake depth measurements. (TIF) [file pone.0111469.s001.tif]

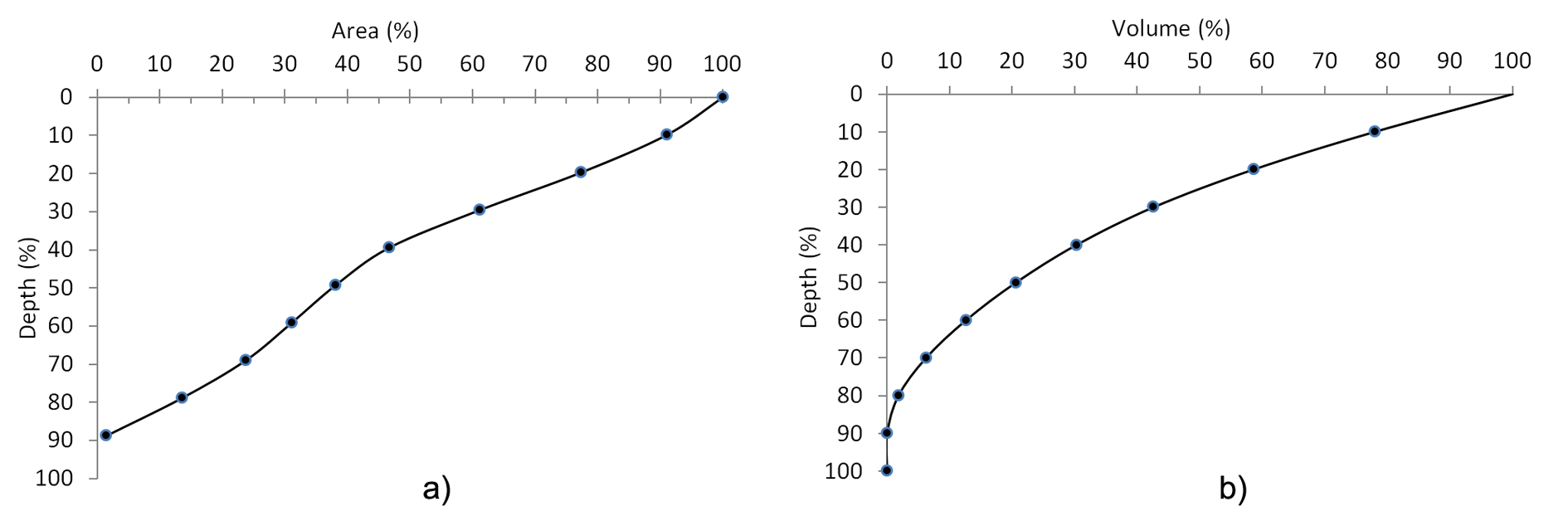

Supplement: Figure S2 — Hypsographic curves of percent total surface (a) and total volume (b). (TIF) [file pone.0111469.s002.tif]
