# Supplementary material for: The Morphometry of Lake Palmas, a Deep Natural Lake in Brazil
Source: PLoS One. 2014 Nov 18;9(11):e111469. doi: 10.1371/journal.pone.0111469 (PMC4236007; doi:10.1371/journal.pone.0111469)
Supplement: Table S1 — Descriptive statistics of limnological variables in wet/warm and dry/mild cold seasons (2011 to 2013). Data from field vertical profiling at 4 sampling sites” from surface to the bottom. (DOCX) [file pone.0111469.s003.docx]

Table S1. Descriptive statistics of limnological variables in wet/warm and dry/mild cold seasons (2011 to 2013). Data from field vertical profiling at 4 sampling sites” from surface to the bottom

|  | Wet/warm | | | | | Dry/mild cold | | | | |
| --- | --- | --- | --- | --- | --- | --- | --- | --- | --- | --- |
| Variables | N | Average | Min | Max | SD | N | Average | Min | Max | SD |
| Z_Sd_ (m) | 17 | 3.8 | 2.1 | 5.9 | 1.0 | 21 | 4.3 | 2.8 | 6.5 | 1.0 |
| Z_eu_ (m) | 17 | 10.9 | 6.3 | 17.7 | 2.8 | 21 | 12.6 | 8.4 | 18.0 | 2.5 |
| Z_mix_ (m) | 20 | 8.4 | 1.0 | 12.0 | 2.5 | 21 | 20.7 | 11.0 | 40.0 | 8.3 |
| Z_eu:_Z_mix_ | 17 | 1.6 | 0.9 | 7.8 | 1.5 | 21 | 0.7 | 0.3 | 1.0 | 0.2 |
| Temperature (°C) | 415 | 26.5 | 21.7 | 31.3 | 2.3 | 442 | 23.2 | 21.5 | 25.6 | 1.2 |
| W_e_ | 20 | 6.5 | 0.0 | 23.6 | 7.2 | 12 | 0.0 | 0.0 | 0.0 | 0.0 |
| Conductivity (µS.cm^-1^) | 415 | 67.2 | 56.0 | 79.0 | 2.6 | 442 | 66.1 | 61.0 | 73.0 | 2.5 |
| DO (mg.L^-1^) | 415 | 4.6 | 0.2 | 9.2 | 2.5 | 442 | 7.3 | 1.7 | 16.5 | 2.1 |

N: number of samples; SD: standard deviation; Z_Sd_: Secchi disk depth; Z_eu_  euphotic depth; Z_mix_ mixing depth; W_e_: effective Wedderburn number.
