# Supplementary material for: The Morphometry of Lake Palmas, a Deep Natural Lake in Brazil
Source: PLoS One. 2014 Nov 18;9(11):e111469. doi: 10.1371/journal.pone.0111469 (PMC4236007; doi:10.1371/journal.pone.0111469)
Supplement: Table S2 — Morphometry of natural lakes in Brazil deeper than 6.0 m. (DOCX) [file pone.0111469.s004.docx]

Table S2: Morphometry of natural lakes in Brazil deeper than 6.0 m.

| Lake | Lake type | Geographical  coordinate | A  (km^2^) | V  (m^3^) | Z_max_  (m) | Z_mv_  (m) | Z_r_  (%) | L_max_  (km) | B_max_  (km) | P  (km) | D_L_ | V_d_ | Ref. |
| --- | --- | --- | --- | --- | --- | --- | --- | --- | --- | --- | --- | --- | --- |
| Palmas (LDRV) | F | 19°25’S 40°15’W | 10.3 | 220.0 x 10^6^ | 50.7 | 21.4 | 1.4 | 7.1 | 1.9 | 51.9 | 4.5 | 1.3 | This study. |
| Dom Helvécio (MDRV) | F | 19°46’S 42°35’W | 5.3 | 59.0 x 10^6^ | 39.2 | 11.3 | 1.5 | 3.1 | 1.2 | 37.7 | 4.6 | 0.9 | 18 |
| Carioca (MDRV) | F | 19°45’S 42°37’W | 0.14 | 0.67 x 10^6^ | 11.8 | 4.8 | 2.8 | 0.57 | 0.4 | 1.7 | 1.3 | 1.2 | 34 |
| Jacaré (MDRV) | F | 19°48’S 42°38’W | 1.0 | 3.8 x 10^6^ | 9.8 | 3.7 |  |  |  | 11.3 | 3.1 |  | 46, 47 |
| Aníbal | F | 19°06’S 42°29’W | 2.79 |  | 6.0 |  |  |  |  |  | 4.3 |  | 47 |
| Gambá | F | 19°47’S 42°35’W | 0.22 |  | 12.0 |  |  |  |  |  | 1.13 |  | 47 |
| Gambazinho | F | 19°47’S 42°34’W | 0.09 |  | 10.0 |  |  |  |  |  | 2.9 |  | 47 |
| Patos (MG) | F | 19°48’S 42°32’W | 1.09 |  | 8.0 |  |  |  |  |  | 2.0 |  | 47 |
| Santa Helena | F | 19°47’S 42°33’W | 0.86 |  | 10.5 |  |  |  |  |  | 2.42 |  | 47 |
| Águas Claras | F | 19°49’S 42°35’W | 0.62 |  | 9.5 |  |  |  |  |  | 2.24 |  | 47 |
| Almácega | F | 19°51’S 42°37’W | 1.30 |  | 7.0 |  |  |  |  |  | 2.44 |  | 47 |
| Barra | F | 19°48’S 42°37’W | 1.94 |  | 7.0 |  |  |  |  |  | 1.9 |  | 47 |
| Palmeirinhas | F | 19°49’S 42°36’W | 0.23 |  | 6.0 |  |  |  |  |  | 2.8 |  | 47 |
| Verde | F | 19°49’S 42°37’W | 0.83 |  | 19.0 |  |  |  |  |  | 2.29 |  | 47 |
| Central | F | 19°38’S 43°53’W | 1.7 | 7.1 x 106 | 7.3 | 4.0 | 0,5 | 1.96 | 1.57 | 6.5 | 1.4 | 1.7 | 48 |
| Baia das Pedras | F | 16°24’S 56°10 W | 0.021 | 0.021 x 106 | 6.2 | 1.0 | 3.8 | 0.27 | 0.10 | 1.1 | 2.1 | 0.5 | 49 |
| Tupé | FF | 03°0.2’S 60°15’W | 0.6 | 1.4 x 10^6^ | 6.0 | 2.1 | 2.2 | 2.5 | 0.25 |  |  |  | 50 |
| Calado | FF | 03°16’S 60°35’W | 8.0 |  | 12.0 |  |  |  |  |  |  |  | 36 |
| Batata | FF | 01°25’S 56°15’W | 18.04 | 39.6 x 10^6^ | 5.5** | 2.19 | 0.11 | 5.00 | 2.10 | 66.2 | 4.40 | 1.2 | 51 |
| Patos (RS) | CL | 31º22’S 51º29W | 9800 | 20000.0 x 10^6*^ | 14.0 | 2.0 |  | 266 | 60 | 959 | 2.7 |  | 52, 53, 54 |
| Mirim | CL | 32º41’S 52º50’W | 560 | 19000.0 x 10^6^ | 18.0 | 5.0 |  | 185 | 40 | 588 | 2.7 |  | 52, 54 |
| Peri | CL | 27°44’S 48°31’W | 5.7 |  | 11.0 | 7.0 |  |  |  |  |  |  | 55 |
| Araruama | CL | 22°52’S 42°12’W | 210 | 610.0 x 10^6^ | 17.0 | 3.0 |  | 40.0 | 13.0 | 331.0 |  |  | 56 |
| Dos Barros | C | 29°55’S 50°22’W | 7.6 | 320 x 10^6^ | 7.5 | 4.2 |  |  |  |  |  |  | 16 |
| Baninho | C | ~ 30°S 50°W | 0.5 | 1.7 x 10^6^ | 6.0 | 3.5 |  |  |  |  |  |  | 16 |
| Taínha | C | ~ 30°S 50°W | 0.5 | 1.7 x 10^6^ | 6.2 | 3.6 |  |  |  |  |  |  | 16 |
| Barro Velho | C | ~ 30°S 50°W | 4.9 | 21.0 x 10^6^ | 8.0 | 4.4 |  |  |  |  |  |  | 16 |
| Moleques | C | ~ 30°S 50°W | 1.1 | 6.1 x 10^6^ | 11.0 | 5.8 |  |  |  |  |  |  | 16 |
| Tarumã | C | ~ 30°S 50°W | 4.2 | 14.0 x 10^6^ | 6.0 | 3.5 |  |  |  |  |  |  | 16 |
| Figueira | C | ~ 30°S 50°W | 7.1 | 37.0 x 10^6^ | 11.0 | 5.8 |  |  |  |  |  |  | 16 |
| São Simão | C | ~ 30°S 50°W | 4.6 | 16.0 x 10^6^ | 6.0 | 3.5 |  |  |  |  |  |  | 16 |

LDRV: Lower Doce River Valley; MDRV: Middle Doce River Valley; MG: Minas Gerais State; RS: Rio Grande do Sul State

Lake type: F: fluvial; FF: fluvial floodplain; C: coastal; CL: coastal lagoon.

A: Lake Area; V: lake volume; Z_max_: Maximum depth; Z_mev_ Mean depth; Z_me_:Relative depth; Lmax: Maximum length; B_max_: Maximum breadth; P: Shoreline length; D_L_: shoreline development index; V_d_: Volume development

* estimated volume according to Hedendorf (1984)
